# Laryngeal Neuroendocrine Carcinomas: A Retrospective Study of 14 Cases

**DOI:** 10.1155/2015/832194

**Published:** 2015-07-15

**Authors:** Yingying Zhu, Liming Gao, Yunxiao Meng, Wenwen Diao, Xiaoli Zhu, Guojun Li, Zhiqiang Gao, Xingming Chen

**Affiliations:** ^1^Department of Otolaryngology-Head and Neck Surgery, Peking Union Medical College Hospital, Chinese Academy of Medical Sciences, Peking Union Medical College, No. 1, Shuaifuyuan, Wangfujing, Dongcheng District, Beijing 100730, China; ^2^Department of Pathology, Peking Union Medical College Hospital, Chinese Academy of Medical Sciences, Peking Union Medical College, No. 1, Shuaifuyuan, Wangfujing, Dongcheng District, Beijing 100730, China; ^3^Department of Head and Neck Surgery, The University of Texas MD Anderson Cancer Center, Houston, TX 77030, USA

## Abstract

Laryngeal neuroendocrine carcinomas (LNECs) are rare and highly heterogeneous which present a wide spectrum of pathological and clinical manifestations. Fourteen patients with histologically demonstrated LNEC were collected and analyzed retrospectively. The 14 cases were classified into 3 subtypes: typical carcinoid in 2, atypical carcinoid in 5, and small cell neuroendocrine carcinoma in 7. The mean survival time of the 14 patients in this study was 112.5 months (95% CI, 81.5–143.6). Surgeries were performed for 2 patients of typical carcinoid, and they were alive with no evidence of recurrence after 24 and 47 months of follow-ups. Patients in the atypical carcinoid group were treated with surgeries and postoperative radiotherapy. After 58.4 months of follow-ups (range: 9–144), 2 patients showed no evidence of disease and 1 was lost to follow-up after 72 months. The other 2 patients died of other unrelated diseases. In the small cell neuroendocrine carcinoma group, a combination of chemotherapy and radiotherapy was applied. The mean survival time was 79.7 months (95% CI, 37.9–121.4), and the 5-year survival rate was 53.6%. In conclusion, the clinical behaviors, treatment protocols, and prognosis are different for each subtype of LNECs.

## 1. Introduction

Laryngeal neuroendocrine carcinomas (LNECs) are rare but are the most common nonsquamous tumors of this organ, which can be divided into 2 broad categories based on their tissue of origin: epithelial and neural. Laryngeal epithelial neuroendocrine neoplasms are all malignant lesions. The major categories of LNECs are typical carcinoid, atypical carcinoid, small cell neuroendocrine carcinoma, and large cell neuroendocrine carcinoma. The neural category is mainly referred to as paraganglioma, which is invariably benign [1]. Since the first report of an atypical carcinoid tumor of the larynx in 1969 by Goldman et al. [2], the larynx has received increasing attention in the potential for development of different neuroendocrine neoplasms there. To date approximately 700 cases of LNECs have been reported in the literatures [3], and yet most of them are case reports. The aim of this study was to improve the understanding of the clinical features by reviewing the literature and analyzing the medical records of patients who were diagnosed with LNEC in our hospital.

## 2. Materials and Methods

Patients diagnosed with LNEC and received treatment in Peking Union Medical College Hospital, China, between 1989 and 2014 were reviewed retrospectively. Available data of gender, age, clinical presentation, tumor site, tumor subtype, treatment, treatment outcome, follow-up, and vital status were extracted from the medical records of patients. Among these, endoscopy, imaging tests, and pathological examination were performed to confirm the location and diagnosis of the mass. The follow-up of the patients ended on February 28, 2015. Overall survival (OS) was defined as the time from first diagnosis to death from any cause or date of last follow-up. Participants who were alive at the end of the study period or lost to follow-up were considered censored. Medical record review for follow-up status of all patients was performed under direct supervision of attending head and neck surgeon. Primary tumor site, clinical stage, treatment, and vital status were reviewed from medical records as assessed between the initial and final patients contacts recorded. All histological samples were reconfirmed by one senior pathologist. Survival analysis was performed using the Kaplan-Meier method, reported as mean with 95% confidence intervals (CI). SPSS software, version 16.0 (SPSS, Inc., Chicago, IL, USA), was used for the statistical analyses. The Ethics Committee of Peking Union Medical College Hospital approved the protocol of this study.

## 3. Results

A total of 14 cases of LNECs were available for analysis. The clinical features are summarized in Table 1. Seven patients were males and 7 were females. The median age at the time of diagnosis was 57 years (range, 16–75). The chief complaints at presentation included hoarseness, sore throat, hemoptysis, a lump sensation, and dyspnea. Two cases were pathologically diagnosed as typical carcinoid, 5 as atypical carcinoid, and 7 as small cell neuroendocrine carcinoma. One of the small cell neuroendocrine carcinoma cases was demonstrated to couple with adenocarcinoma. The mean survival time of all 14 patients in this study was 112.5 months (95% CI, 81.5–143.6). The 2-year and 5-year survival rate in this series were 84.4% and 73.9%, respectively.

Two patients with typical carcinoid showed supraglottic lesions. Microsurgery was performed for one case, as the tumor was confined to one side of arytenoid cartilage. Total laryngectomy and neck dissection were performed in the other patient with more extensive lesions. Both patients were alive with no evidence of recurrence after 24 and 47 months of follow-up, respectively.

Among 5 patients with atypical carcinoid, 3 patients showed supraglottic lesions, and 2 showed glottic lesions in the larynx. The treatment included surgery and radiotherapy. Four patients underwent laryngectomy with reservation of laryngeal function, and total laryngectomy was performed for the other patient with more extensive lesions. Neck dissection was performed concurrently in 4 patients because of lymph node metastases. Postoperative radiotherapy was applied in 2 patients. As a result, 2 patients showed no evidence of disease and 1 was lost to follow-up after 72 months. The other 2 patients died of rectum carcinoma and nephropathy, respectively, which were unrelated to the primary diseases. The mean follow-up time was 58.4 months (range: 9–144).

Among all 7 patients with small cell neuroendocrine carcinoma, 5 patients showed supraglottic lesions, 1 showed subglottic lesion, and the other showed glottic lesion in the larynx. At the presentation, 4 patients developed cervical metastases either unilaterally or bilaterally, but no distant metastases were found. All patients received chemotherapy (platinum and VP16) and radiotherapy (a median total dose of 58 Gy). Complete remission occurred in 4 patients and partial remission occurred in 3 cases. Among the 4 patients with complete remission, to date, 3 patients survived for 49, 72, and 129 months, respectively. Another patient presented distant metastases 16 months later and died within 24 months. Two of the patients with partial remission developed distant metastases and died within 5 and 36 months after diagnosis, respectively. The other one was diagnosed as small cell neuroendocrine carcinoma combined with adenocarcinoma and lost to follow-up after 30 months. The mean survival time of the 7 patients was 79.7 months (95% CI, 37.9–121.4). The 5-year survival rate was 53.6%.

## 4. Discussion

LNECs account for approximately 1% of all laryngeal neoplasms [4]. The different subtypes show a variety of different natural history, treatment, and radically different outcomes. They occurred mainly in elderly men; and smoking has been recognized as one of risk factors. The atypical carcinoid is the most frequent subtype, followed by the small cell neuroendocrine carcinoma, typical carcinoid, and the large cell neuroendocrine carcinoma [5]. The overall average age of the current series was 52.7 years, while in the current series 3 patients were less than 30 years old and no significant difference was based on sex. The small cell neuroendocrine carcinoma accounted for the largest proportion (7/14) in our series, which could be partially due to the limited number of cases.

According to the World Health Organization classification, LNECs were divided into three groups: typical carcinoid, atypical carcinoid, and small cell neuroendocrine carcinoma. Laryngeal large cell neuroendocrine carcinoma was classified together with atypical carcinoid group [6]. However, more recent study suggested that large cell neuroendocrine carcinoma should be separated from atypical carcinoid as a distinct entity because of their much greater aggressive potential [7]. In this study, we reported 14 cases including typical carcinoid, atypical carcinoid, and small cell neuroendocrine carcinoma. The clinical features are different for each LNEC subtype.

Typical carcinoid presents as submucosal masses which may be either polypoid or nodular. The clinical course is slow owing to the benign biological behavior. Conservative surgical excision is considered to be the best choice for typical carcinoid of larynx. Endolaryngeal microsurgery can be an ideal choice for limited tumors. Elective neck dissection is unnecessary [8]. The 5-year survival rate of typical carcinoid of larynx was 48.7% in a large series report [9]. In our study, surgery was performed in 2 patients who were alive with no evidence of recurrence after 24 and 47 months of follow-up, respectively.

Atypical carcinoid manifests as nodular submucosal masses with necrosis. The mainstay of treatment is surgical excision. Partial or total laryngectomy is recommended depending on the size and extension of the tumor considering their highly aggressive nature [10]. Elective neck dissection should be performed concurrently in view of high incidence of lymph node metastases. Gillenwater et al. [11] reported that a few patients with atypical carcinoid tumors responded to radiotherapy and chemotherapy; and the 5-year survival rate of this tumor was approximately 46.7% [9]. In our study, surgery was performed in all cases, which varied from partial laryngectomy to total laryngectomy, and neck dissection was performed in the cases of lymph node metastases. Postoperative radiotherapy was applied in 2 patients. Since most of cases (3/5 cases) were of censored data, therefore the survival estimate could not be taken in this group.

Small cell neuroendocrine carcinoma is the most lethal tumor of the larynx, which develops rapidly. More than 90% of these patients have metastatic disease in its clinical course, and the most common sites of metastatic spread are the cervical lymph node, liver, lung, and bone [12]. In the current series, more than half of the patients developed cervical metastases at the presentation. The therapeutic protocol of a combination of local radiotherapy and chemotherapy has been generally accepted. Baugh et al. [13] reviewed the various modalities that have been used to treat this cancer. The combination of radiation therapy and adjuvant chemotherapy resulted in a median survival of 55 months, representing significantly longer survival than with any other treatment regimen. There is no consensus on its appropriate chemotherapy protocol by now. The main protocol used in our hospital was combination of platinum and etoposide. In our series, complete remission rate was 57% (4/7) and efficiency rate was 100% (7/7). The survival time in complete remission cases was significantly better than that of the partial ones, which demonstrated that the response to chemotherapy is closely related to the prognosis. Although radiation alone might not improve survival, it is successful in controlling primary lesion, which was consistent with our study.

Surgical excision is limited even in the early lesion of small cell neuroendocrine carcinoma, which should be avoided [14]. One patient in our study was diagnosed as having adenocarcinoma preliminarily, so surgery was performed at the beginning, and the patient developed relapse after 12 months. The final diagnosis was acquired as small cell neuroendocrine carcinoma is coupled with adenocarcinoma by endolarynscopic biopsy. This should be a lesson we may learn from this study.

The prognosis of small cell neuroendocrine carcinoma of larynx was very poor; 5-year survival rate was less than 10% [12, 15]. In the current study, the mean survival time was 79.7 months (95% CI, 37.9–121.4). The 5-year survival rate was 53.6% which was much higher than that in literatures. The reason might be due to the small sample size in our study; thus it needs larger studies with longer follow-up time to confirm our finding.

Laryngeal large cell neuroendocrine carcinoma is a newly described clinicopathological entity, and therapy is not standardized for this tumor. Similarly to the situation in cases from the lung, considerable debate has emerged as to whether this tumor should be treated as small cell neuroendocrine carcinoma or in different ways [1]. However, this type was not included in the current series.

In conclusion, LNECs are rare types of nonsquamous neoplasms of the larynx. Owing to their variation, clinical behaviors, treatment protocols, and prognosis of LNECs are different for each subtype. Therefore, it is necessary for pathologists and clinicians to be familiar with each type of neuroendocrine neoplasms of larynx so that they can treat these diseases appropriately.

## Figures and Tables

**Table 1:** Clinical features of 14 cases with LNEC.

Case number	Age (years)	Sex	Tumor site of larynx	Tumor type	Initial treatment	Disease-free period (months)	Follow-up (months)/vital status
1	61	F	Supraglottic	TC	Endolaryngeal microsurgery	47	47/NED

2	60	F	Supraglottic	TC	TL and ND	24	24/NED

3	75	M	Glottic	AC	PL and ND	19	19/DOC

4	62	M	Supraglottic	AC	PL and ND	144	144/NED

5	66	F	Supraglottic	AC	PL, ND, and radiotherapy	48	48/DOC

6	30	F	Supraglottic	AC	PL	72	72/lost to follow-up

7	66	M	Glottic	AC	TL, ND, and radiotherapy	9	9/NED

8	53	F	Supraglottic	SCNEC	Chemotherapy and radiotherapy	72	72/NED

9	66	M	Supraglottic	SCNEC	Induction chemotherapy, radiotherapy, and adjuvant chemotherapy	20	36/DOD

10	27	F	Subglottic	SCNEC	CRT and chemotherapy	129	129/NED

11	50	M	Supraglottic	SCNEC	Chemotherapy and radiotherapy	49	49/lost to follow-up

12	54	M	Supraglottic	SCNEC	Induction chemotherapy, radiotherapy, and adjuvant chemotherapy	16	24/DOD

13	52	M	Supraglottic	SCNEC and adenocarcinoma	PL, ND, chemotherapy, and radiotherapy	30	30/lost to follow-up

14	16	F	Glottic	SCNEC	Chemotherapy and radiotherapy	5	5/DOD

TC: typical carcinoid; AC: atypical carcinoid; SCNEC: small cell neuroendocrine carcinoma; TL: total laryngectomy; PL: partial laryngectomy; ND: neck dissection; CRT: concurrent radiochemotherapy; NED: no evidence of disease; DOC: dead of other causes; and DOD: dead of disease.

## Abbreviations

LNECs: Laryngeal neuroendocrine carcinomas
OS: Overall survival
CI: Confidence interval.
